# Modulating the Optoelectronic Properties of Silver Nanowires Films: Effect of Capping Agent and Deposition Technique

**DOI:** 10.3390/ma8115405

**Published:** 2015-11-11

**Authors:** D. Lopez-Diaz, C. Merino, M. M. Velázquez

**Affiliations:** 1 Departamento de Química Física, Facultad de Ciencias Químicas, Universidad de Salamanca, Salamanca 37008, Spain; dld@usal.es; 2 GRAnPH Nanotech, Grupo Antolín Ingeniería SA, Burgos 09007, Spain; cesar.merino@grupoantolin.com

**Keywords:** silver nanowires, spray-coating, Langmuir-Schaefer, transparent conductive electrodes

## Abstract

Silver nanowires 90 nm in diameter and 9 µm in length have been synthesized using different capping agents: polyvinyl pyrrolidone (PVP) and alkyl thiol of different chain lengths. The nanowire structure is not influenced by the displacement of PVP by alkyl thiols, although alkyl thiols modify the lateral aggregation of nanowires. We examined the effect of the capping agent and the deposition method on the optical and electrical properties of films prepared by Spray and the Langmuir-Schaefer methodologies. Our results revealed that nanowires capped with PVP and C8-thiol present the best optoelectronic properties. By using different deposition techniques and by modifying the nanowire surface density, we can modulate the optoelectronic properties of films. This strategy allows obtaining films with the optoelectronic properties required to manufacture touch screens and electromagnetic shielding.

## 1. Introduction

Thin solid films with a combination of high optical transparency and low electrical resistance are crucial for manufacturing modern devices such as photovoltaic cells, touch panels, light-emitting diodes (LEDs), transparent heaters, and many other devices [1,2]. The development of new materials with appropriate properties is mostly dictated by the requirements of each application [1]; however, low optical absorption and high electrical conductivity always constitute significant requisites. In recent years, Indium Tin Oxide (ITO) has dominated the field of transparent conductive electrodes (TCEs) owing to its excellent optoelectronic properties, which can be tuned depending on the needs of each application [3]. Despite its excellent properties, ITO suffers important limitations such as the scarcity of Indium, a high-cost fabrication process, and the brittleness of its electrodes. Moreover, when ITO is deposited onto flexible substrates, it gives rise to bending fatigue; therefore, a substitute for the ITO electrode with similar electrical and optical properties is clearly needed [4].

In order to replace ITO, several alternative materials, such as carbon nanotubes [5,6], graphene films [7,8], conducting polymers, and metal nanowires [1,9] have been proposed. However, some drawbacks have to be solved. In the case of polymers, their chemical stability is the main problem [10]. Carbon nanotubes present good electrical conductivity, but the transparency is too low, while the opposite behavior was found for electrodes built with graphene, since they present good transparence, although the sheet resistance decreases due to edge defects [11]. Among these materials, metal nanowires and in particular, silver nanowires, are especially promising since silver exhibits one of the highest conductivities (6.3 × 10^7^ S·m^−1^) [12]. For manufacturing transparent electrodes with metal nanowires, the challenge is to obtain an optimal network with the desirable values of both transparency and sheet resistance. It is well known that transparency and sheet resistance (*R*_s_) have opposite dependences on nanowire density; therefore, it becomes necessary to optimize the nanowire orientation along its surface density for each application. In addition to density, resistance is highly influenced by the contact resistance between crossing nanowires [13], which depends on the nanowire arrangement, capping agent, and nanowire structure. Thus, it has been previously reported that longer and thinner nanowires reduce resistance while increasing film transparency [14,15,16]. This behavior is due to the low density needed to achieve low resistance on the film. Therefore, it is necessary to modify the nanowire structure to optimize the optoelectronic properties of films.

A crucial issue in building metal nanowire devices is the transfer process from solutions to solid substrates. This is because alignment and controlled positioning are highly desirable. Several techniques such as spin coating [17] and drop casting [13] have been used to prepare films of hydrophilic silver nanowires (AgNW) synthesized by the polyol method and using PVP as stabilizer. Spin coating and drop casting methodologies present several disadvantages, since water evaporation leaves discontinuous films with typical coffee rings that influence the quality of AgNW films [18,19].

We expect that it is possible to accomplish a controlled positioning of nanowires using suitable stabilizing agents combined with the appropriate deposition techniques. With this objective in mind, we analyze the effect of stabilizing agent on the structural properties of silver nanowires by replacing the polymer typically used in the synthesis of silver nanowires, PVP, by alkyl thiol molecules. We chose alkyl thiol molecules as a stabilizing agent because the sulfur contained in thiol groups forms a strong chemical bond with Ag, rending more homogeneous layers of capping agents than PVP [20,21]. We expect that this structural change could improve the electrical resistance and optical absorption of films prepared with these nanowires. On the other hand, the replacement of PVP molecules by alkyl thiol leads to hydrophobic nanowires that can form stable Langmuir monolayers. These monolayers can be transferred onto solids by a controlled method such as the Langmuir-Schaefer (LS) technique. The LS deposition methodology consists of the horizontal transfer process of Langmuir films from the air-water interface onto solids. This technique allows continuous variation of particle density, spacing, and arrangement by compressing or expanding the film by using barriers [22]. Consequently, the method offers the possibility of preparing reproducible films with the control of interparticle distance necessary for the manufacture of good quality AgNW films. Moreover, since the substrate is not dipped in an aqueous subphase in this method, the undesirable processes induced by water evaporation are minimized.

Finally, we compare the results obtained by LS (mid-scale methodology) with those obtained by scalable methods such as spray coating. We chose this technique because is a cheap, simple, compatible with low temperature methods, and was successfully used for the deposition of new materials such as graphene oxide nanocomposites [8] and carbon nanotubes [6].

## 2. Results and Discussion

### 2.1. Characterization of Silver Nanowires

Silver nanowires exhibit a wealth of optical properties directly related to the Surface Plasmon Resonance (SPR). SPR originates from the coherent oscillation of the conduction band electrons induced by interaction with the electromagnetic field and mainly depends on nanowire geometry [23]. Therefore, the UV-Vis spectrum of nanowire solutions are often used to extract qualitative information about the nanowire structure. Accordingly, we have recorded the UV-Vis spectra of AgNW dissolved in ethanol (PVP-AgNW) and in chloroform (alkyl thiol-AgNW). The spectra are plotted in Figure S1 of the Supplementary Material (SM) and they present an intense band at around 400 nm. This band is characteristic of the transversal SPR of AgNW, and the position of the maximum is almost independent of the nature of molecules used as capping agents. Taking into account that the maximum position has been related to the nanowire diameter [22], our results seem to indicate that the replacement of PVP by alkyl thiol does not modify it. To confirm this, the diameter and length of the nanowires were calculated from Field Emission-Scanning Electron Microscopy (FE-SEM) images (Carl Zeiss Sigma, Jena, Germany). Representative FE-SEM images are in Figure S2 of the SM. The images correspond to spin-coated AgNW deposited onto Si/SiO_2_ at 1000 rpm. The nanowire solutions were prepared on ethanol or chloroform depending of the nanowire nature. The solution concentration was kept constant at 0.2 mg/mL. We have analyzed the FE-SEM images and results are summarized in Table 1 and Table S1. Details of the statistical analysis are included in Section 1 of the SM, Figures S2–S4.

**Table 1:** Length and diameter values of nanowires with different capping agents. Nanowire Length (L); Full Width at Half Maximum (FWHM).

Capping Agent	*L*/µm	FWHM/µm	*D*/nm Single AgNW	Percentage of Single AgNW (%)	*D*/nm AgNW Aggregates	Percentage of AgNW Aggregates (%)
PVP	9.2 ± 0.3	9 ± 1	83 ± 2	28.6	155 ± 16	50.0
					250 ± 5	21.4
C8	8.8 ± 0.7	12 ± 2	94 ± 6	38.4	160 ± 12	50.8
					254 ± 5	10.8
C12	9.2 ± 0.9	14 ± 4	89 ± 7	35.8	157 ± 7	32.8
					256 ± 12	31.4
C18	9.2 ± 0.5	12 ± 2	100 ± 2	11.3	160 ± 8	54.7
					327 ± 46	34.0

From the results displayed in Table 1, it is possible to conclude that the nanowire length, *L*, is almost independent of the capping agent and the averaged value is 9.1 ± 0.2 µm. On the other hand, the distribution of nanowire length can be fitted to a Gaussian function, see Figure S3 of the SM, and the width, measured by the full width at half maximum (FWHM), was taken as an estimate of the polydispersity degree. The FWHM values are collected in Table 1 and show that the polydispersity degree is slightly higher for nanowires capped with alkyl thiol than for PVP-capped nanowires. The FE-SEM images show the presence of single and laterally fused nanowires (Figure S2). The diameter of laterally fused nanowires is compatible with interactions between two nanowires, referred to as dimer hereafter, and three nanowires, trimer. Moreover, the single nanowire diameter values seem to be independent of capping agent, and the average value found was 92 ± 7 nm. From our results, the L/D value found for a single nanowire was 100. This is an acceptable value for preparing good performance transparent conductors [24]. Finally, the percentage of single nanowires decreases when the number of C atoms of the alkyl thiol chain increases. This is an expected behavior if one considers that when the chain length of the capping agent increases, attractive interactions between capping agents also increase, promoting lateral aggregation.

### 2.2. Silver Nanowire Films

After nanowire characterization, we found that nanowires have a good *L*/*D* ratio for reaching competitive optoelectronic properties; therefore, the next step was to study the effect of the deposition technique on the optoelectronic properties of films. The two deposition methodologies selected were spray coating and the Langmuir-Schaefer technique.

#### 2.2.1. Spray-Coating Films

Prior to obtain the nanowire films by the spray coating method it is mandatory to identify the best experimental conditions. With this objective in mind, we have checked the influence of the backpressure, nozzle to polycarbonate (PC) distance and substrate temperature on the optoelectronic properties of films. We analyze the effect of these parameters because they have proved to be the most critical ones in the performance of the spray process [25]. The best conditions of reproducibility and homogeneity were obtained at the backpressure value of 29.7 psi, nozzle-PC distance of 10 cm and substrate temperature of 120 °C. These parameters are in excellent agreement with those obtained by other authors [25]. With these conditions, several AgNW films were prepared over PC substrates of 2.5 cm × 2.5 cm and the resistance and transparency of films were measured. The next step was to analyze the effect of the nature of the capping agent on the optoelectronic properties of films. Table 2 and Table S2 collect transmittance (*T*, %) and *R*_s_ values of films prepared with different nanowire mass. In Figure S5 of the Supplementary Material the *R*_s_ and *T* (%) values are plotted against the mass of nanowire deposited onto the substrate.

**Table 2:** Resistance and Transmittance values of AgNW films built by spray coating. Sheet resistance (*R*_s_); Transmittance (*T*).

Capping Agent	*m*/mg	*R*_s_/Ω·sq^−1^	*T* (%)
PVP	2.25	192	63.2
	3.00	22	52.8
C8	2.25	19	73.4
	3.00	22	65.2
C12	2.25	1.5 × 10^5^	76.4
	3.00	8.5 × 10^5^	73.0
C18	3.00	Insulator	56.0
	4.50	Insulator	40.3
	6.00	2.0 × 10^4^	28.3

**Figure 1:**
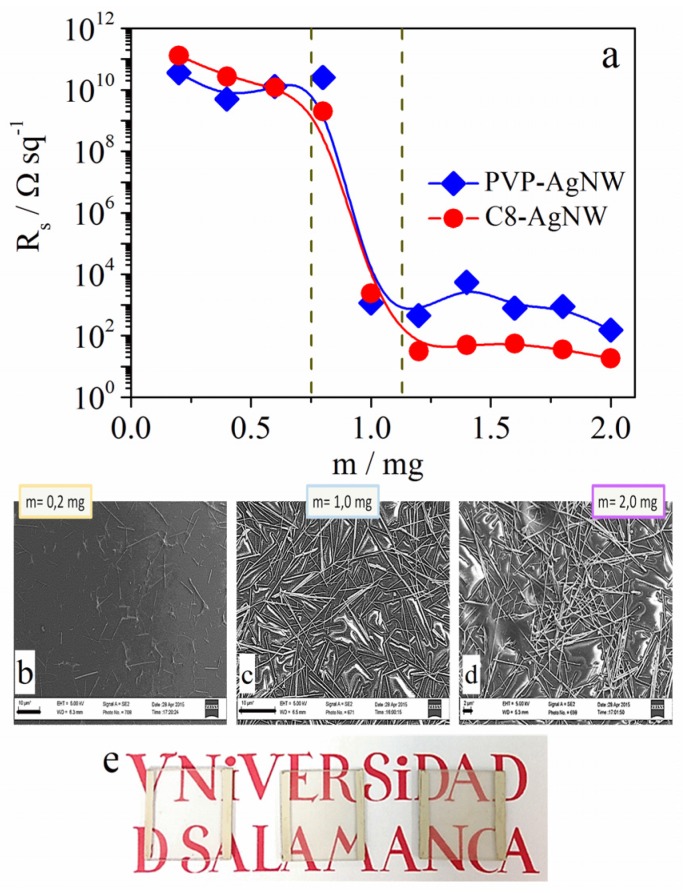
(**a**) Variation of *R*_s_ with the nanowire mass: PVP-AgNWs (Diamonds) and C8-AgNWs (circles); (**b**–**d**) Field Emission-Scanning Electron Microscope (FE-SEM) images and pictures (**e**) of the C8 AgNWs films of different sprayed mass values. Scale length in figures b, c, and d corresponds to 10 µm, 10 µm and 2 µm, respectively.

From results in Table 2, it is possible to conclude that *R*_s_ and *T* (%) decrease when the density of nanowires increases. On the other hand, *R*_s_ values of films built with nanowires stabilized with C12 and C18 alkyl thiols are too high even at high surface concentrations, and consequently, they do not seem to be a good choice for reaching good quality for transparent and conductor devices. As such, we will focus on silver nanowires capped with PVP (PVP-AgNW) and with C8 (C8-AgNW) because they exhibit better properties. In both systems, *R*_s_ reaches good values; however, the transparency has to be improved. Therefore, it is necessary to reduce the nanowire density by preparing films with a mass below 2 mg. The variation of resistance with the sprayed mass is plotted in Figure 1a. FE-SEM images of films and pictures of electrodes were also taken, and representative images are collected in Figure 1b–e. For more details, see Figures S6 and S7 in Section 2.2 of SM.

The variation of *R*_s_ with the nanowire mass shows three different regions. When the nanowire mass is below 0.75 mg, *R*_s_ is too high. In this region, the FE-SEM image in Figure 1b shows that nanowires do not have enough contact with each other and consequently, the electric resistance is very high. In this region, the transparency is also high as can be observed in the picture of the left electrode in Figure 1e. The FE-SEM images and pictures in Figure 1e correspond to the same films. The second region, between 0.75 mg and 1.2 mg corresponds to the transition region in which the contact between nanowires started, as can be seen in Figure 1c. The existence of several contacts is responsible for the drastic decrease of *R*_s_. The third region corresponds to nanowire mass above 1.2 mg. In this region, the *R*_s_ value remains almost constant. FE-SEM images show that above this surface concentration, nanowires have acquired a stable arrangement in which all nanowires are connected. However, due to the increase of nanowire mass, the film transparency decreases (Figure 1e). Results also showed that the saturation values of *R*_s_ are higher for PVP-AgNW than for C8-AgNW. Taking into account that the nanowire geometry is quite similar, this fact suggests that the effects are due to the nature of the capping agent.

**Figure 2:**
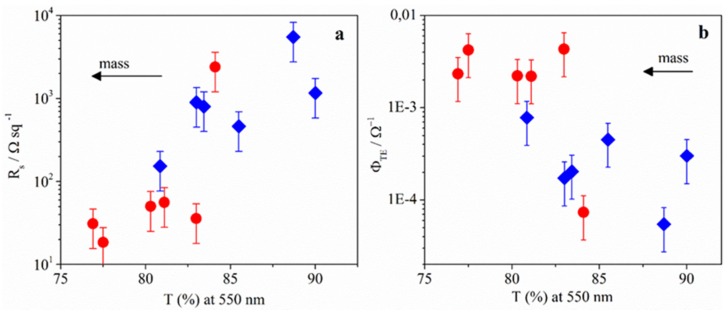
(**a**) Variation of R_s_ and (**b**) the Haacke parameter Φ_TE_ with transmittance for PVP-AgNWs (Diamonds) and C8-AgNWs (circles). Arrows represent the direction in which the mass of AgNWs deposited onto the substrate increases.

To quantify changes in film transparence, we have measured the optical transmittance of films. Figure 2a shows the plot of *R*_s_ *versus* *T* (%) at 550 nm wavelength, for films built with different nanowire surface density. Arrows in Figure 2a,b point to the direction in which the nanowire mass increases. Only those of electrodes with *R*_s_ values below 10^4^ Ω·sq^−1^ were considered in Figure 2a.

As expected, films with the highest nanowire mass present the lowest sheet resistance and transparency. On the other hand, films with transmittance values around 80%–85% present R_s_ values lower for C8 than for PVP. This fact indicates better optoelectronic properties for silver nanowires coated with C8 than with PVP. In order to compare the quality of transparent electrodes, the commonly used figure-of-merit is *Φ*_TE_ [4], defined by Haacke (Equation (1)). In Figure 2b, *Φ*_TE_ values are plotted *vs.* *T*.

(1) ΦTE=T10RS

The best optoelectronic properties correspond to the highest *Φ*_TE_ values [4]. Thus, in our systems, the best values correspond to C8-AgNW films. To interpret this behavior we consider theoretical results recently published [24]. These results demonstrated that for polydisperse networks, a slight increase in the proportion of nanowires with a high *L*/*D* ratio drives a dramatic reduction of the *R*_s_ [24]. This seems to be the situation in our systems, since the population of single nanowires is higher for C8-AgNW (39%) than for PVP-AgNW (29%)—see Section 2.1. Finally, the optimum value found for *Φ*_TE_ was 0.0043 Ω^−1^ for T = 83%. These values are close to those previously reported for thin metal films [26].

#### 2.2.2. Langmuir-Schaefer Films

Electronic Microscopy images of films obtained by spray-coating show a random distribution of nanowires. These films present good optoelectronic properties; however, they could be improved if nanowires were ordered in preferential directions. The Langmuir-Schaefer methodology allows continuous variation of the particle density, spacing, and arrangement; therefore, it can be used to achieve different patterns without a defined template [27]. Accordingly, we expect that the LS methodology provides films with specific nanowire orientation, improving its optoelectronic properties. Since nanowires capped with PVP and C8 led to the best optoelectronic properties in films prepared by spray, we will focus our interest on these systems, although for comparative purposes some C18-AgNW films were prepared. On the other hand, since PVP-AgNWs are water-soluble materials, it was not possible to obtain stable Langmuir monolayers.

Taking into account that nanoparticles at the air-water interface can exist in different 2D states [28,29], prior to the LS deposition, it is necessary to study the surface properties of the Langmuir monolayer. This allows the selection of the surface state to be transferred from the air-water interface to the solid. The characterization of surface states is often carried out in terms of the surface compressional modulus, ε [30]. This parameter is calculated from the surface pressure isotherms and the Equation (2).

(2) $$\text{ε}=\text{Γ}{\left(\text{δ π}/\text{δ Γ}\right)}_{T}$$

**Figure 3:**
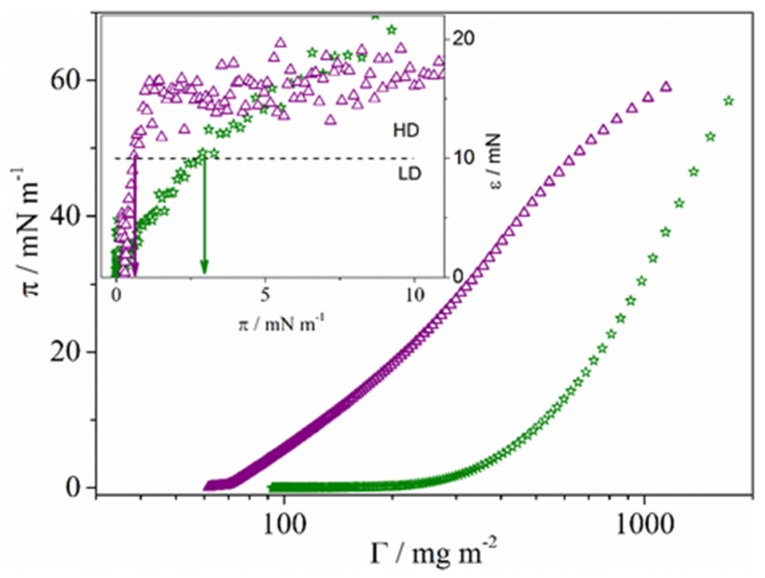
Surface pressure isotherms as a function of the nanowire mass for C8-AgNWs (stars) and C18-AgNWs (triangles). The variation of the surface compressional modulus with the surface pressure is represented in the inset.

In this equation, π and Γ represent the surface pressure and concentration, respectively, expressed in terms of nanowire mass. The C8 and C18 Ag-NW surface pressure isotherms are presented in Figure 3. The variation of the compressional modulus with surface pressure is plotted in the inset of Figure 3.

**Figure 4:**
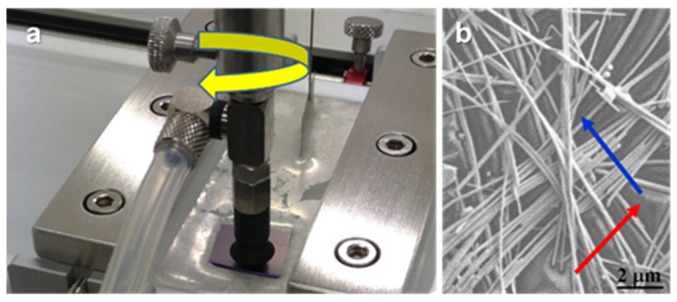
(**a**) Langmuir-Schaefer deposition procedure; (**b**) FE-SEM image of the C8-AgNW bilayer obtained by the Langmuir-Schaefer (LS) methodology. Arrows indicate the orientation of the first (red), and second layers (blue).

As can be seen, the isotherm morphologies are quite similar to those for surfactant molecules [30,31,32] and nanoparticles [28,29,33]. Thus, surface pressure and compressional modulus values close to zero correspond to small nanowire surface concentration. In this region, referred to as the low surface density state (LD), nanowires are randomly orientated. When the surface density increases, the surface pressure and compressional modulus, *ε*, increase. Compressional modulus values above 10 mN·m^−1^ correspond to films highly packed; we referred to this state as the high surface density state (HD) [32]. As can be observed in the inset of Figure 3, the LD state is reached at a lower surface pressure value for AgNW-C18 monolayers (0.5 mN·m^−1^) than for C8-AgNW (3 mN·m^−1^). This is likely due to stronger attractive interactions between the hydrocarbon chains of nanowires capped with C18 than for those capped with C8. If the surface pressure is further increased, nanowires aggregate in a continuous film, see Figure S8 of the SM. Since nanowire aggregation reduces the film transparency, we transfer monolayers at the LD and HD states by using the Langmuir-Schaefer methodology. To manufacture the AgNW networks, a second layer in which nanowires are oriented perpendicular to the first layer was also deposited. In each experiment, the monolayer surface pressure remains constant at a given value. To clarify experiments, Figure 4a shows the image of the deposition procedure. Figure 4b shows the FE-SEM image of a film built with C8-AgNW nanowires at the surface pressure of 15 mN·m^−1^. Those obtained at different surface pressures are collected in Figure S9 of the SM. Arrows in Figure 4b indicate the orientation of the first (red arrow), and second layers (blue arrow).

**Figure 5:**
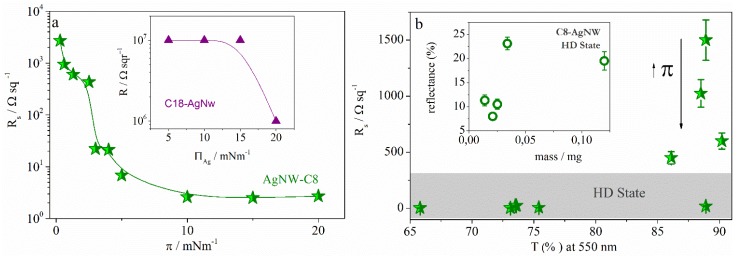
Variation of R_s_ with (**a**) the surface pressure and (**b**) the transmittance for electrodes manufactured with C8-AgNWs (stars). The inset in figure (**a**) represents the variation of R_s_ with the surface pressure for C18-AgNWs (triangles). Inset in (**b**) represents the variation of the reflectance with the C8-AgNWs mass (circles). High Surface Density (HD).

We measured the R_s_ of films thus prepared. Figure 5a shows the variation of the sheet resistance with the surface pressure of the Langmuir monolayer precursor of the LS film.

Results show that C18-AgNW films are an insulator even at high surface concentrations while AgNW-C8 films present two different trends, depending on the transferred state: the LD or the HD state. To analyze this behavior, we plotted *R*_s_ *vs.* *T* (%) in Figure 5b. Results in Figure 5b show that monolayers at the LD state (π < 2 mN·m^−1^) present the highest R_s_ values, and R_s_ decreases as the surface pressure of the Langmuir monolayer increases, while the transparency remains almost unaltered at a higher value (~88%). This is an interesting behavior because one can modulate the resistance of films by modifying the surface concentration of the Langmuir monolayer at the LD state while the transmittance value remains almost constant. The trend is the opposite when films are prepared with HD monolayers. In this case, the sheet resistance remains almost constant at 8 Ω·sq^−1^ while the transmittance value ranges from 65% to 89%. This means that a given nanowire network is established for high-density films. In this network, the R_s_ value remains almost constant while the transmittance decreases as the surface density increases. It is well established that dense nanowire films present high light scattering, which influences the films transparency. Therefore, we have measured the scattered light (reflectance) of films of different nanowire mass. The reflectance values are represented in the inset of Figure 5b. We estimated the mass deposited on the electrodes from the variation of the surface pressure during the deposition process using the surface pressure-concentration isotherms. From the surface concentration thus obtained, and the covered area value (6.25 cm^2^), the mass deposited onto the solid was calculated. Our data show a good correlation between the reflectance values and the mass adsorbed on the solid, confirming the above assumption. The behavior observed in films prepared by transferring Langmuir monolayers at the HD state presents interesting applications because it allows for the tuning of the optical properties of films. It was reported that the large scattering of light can provide better absorption of light in solar cells, which leads to higher efficiencies [13,34]. Therefore, our methodology can be successfully used to modify the scattering of light in these devices.

**Figure 6:**
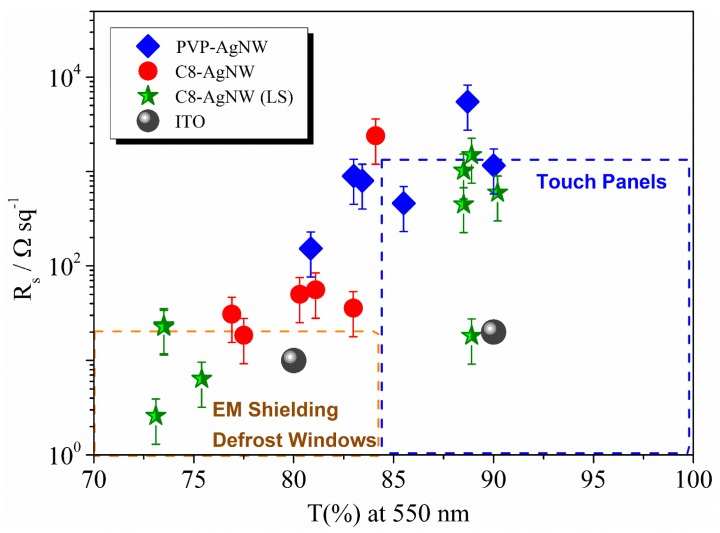
Variation of *R*_s_ with *T* (%) for films obtained with different nanowires and deposition methodologies: PVP-AgNWs Spray (diamonds), C8-AgNWs by Spray (circles) and C8-AgNWs by LS (stars). Gray circles correspond to representative values for Indium Tin Oxide [4,35].

In summary, LS methodology allows tuning of the R_s_ values between 10^3^ and 10 Ω·sq^−1^, keeping the transmittance value close to 88% by transferring LD monolayers. Moreover, it is possible to modulate the values of transparency between 65% and 89%, keeping the R_s_ value around 8 Ω·sq^−1^ when high surface density monolayers are transferred onto the substrate. Finally, for comparative purposes, in Figure 6 the optoelectronic properties of AgNW films obtained in this work are plotted together with those built with ITO. We have selected optoelectronic properties required for the manufacture of touch screens, electromagnetic shielding, or defrosting windows. The range of variation of the properties required for each application is represented as a box in Figure 6. We also plot in Figure 6 two representative values corresponding to ITO films [4,35].

Results in Figure 6 show that electrodes manufactured with nanowires stabilized with PVP or octyl thiol can be employed as substitutes for ITO as components of devices such as touch screens [25], electromagnetic shielding, and defrosted windows [1]. Moreover, we can modulate the optoelectronic properties by modifying the capping agent and the deposition methodology to achieve the requirements needed in each application.

## 3. Experimental Section

### 3.1. Synthesis of Silver Nanowires

Hydrophilic silver nanowires synthesized according to the polyol method were provided by GRAnPH Nanotech. This procedure uses ethylene glycol as solvent and reducing agent and polyvinyl pyrrolidone (PVP, 55kDa) as stabilizer [36]. According to the nature of the capping agent PVP, nanowires present a hydrophilic nature and can be easily dispersed in water and alcohol solvents. The hydrophobic AgNWs were synthesized by replacing the polymer PVP with alkyl thiol molecules of different chain length, referred to as C*_n_*-SH (*n* = 8, 12, 18). The surface modification is achieved through the surface ligand exchange procedure reported by Tao [37]. In this procedure, the same volumes of C*_n_*-SH solutions (*n* = 8, 12, 18), 100 μM dissolved in chloroform, were mixed with silver nanowires in ethanol. The mixture was sonicated for 5 min in a commercial bath to remove PVP and to favor mixing of the reactants. Finally, the reaction was maintained overnight and then centrifuged at 3000 rpm for 15 min. Beige silver nanowires were collected and dispersed in chloroform and then centrifuged at 3000 rpm in order to separate nanowires capped with thiols. The supernatant solution contains the excess thiol molecules and PVP displaced by the thiols. Nanowires thus obtained were dissolved in chloroform and centrifuged at 3000 rpm to remove the unbounded thiol. This procedure was repeated at least six times obtaining a final opaque gray solution of purified nanowires.

Millipore Ultra-pure water, prepared using a combination of RiOs and Milli-Q systems from Millipore (Billerica, MA, USA), was used as subphase in the Langmuir trough. Chloroform (PAI, filtered) supplied by Sigma Aldrich (St. Louis, MO, USA) was used to prepare the spreading solutions and reagents. PVP, ethylene glycol, and alkyl thiol molecules were supplied by Sigma-Aldrich (St. Louis, MO, USA).

In order to build the thin films, AgNWs were deposited onto Lexan Polycarbonate (PC) substrates supplied by Sabic Plastic (Pittsfield, MA, USA). We chose this solid substrate because it is widely used in TCEs for the automotive sector and is one of the most used plastic substrates in flexible applications jointly with Polyethylene Terephthalate (PET). For nanowire characterization by FE-SEM, AgNWs were deposited on an As-doped silicon wafer (100) with 300 nm of dry thermal SiO_2_. This wafer was supplied by CENER (Pamplona, Spain).

### 3.2. Experimental Methods

Two different deposition methodologies, spray-coating and Langmuir-Schaefer, were selected. In all cases, we use PC square substrates of 2.5 cm × 2.5 cm. The substrates were washed with ethanol, water, and dried by pumping nitrogen. To activate the substrate surface, it was exposed in an ozone chamber for 10 min. The ozone chamber used was a UV/ozone Procleaner^TM^ Plus model from BIOFORCE Nanosciences.

For films obtained by air-spraying coating, hydrophilic and hydrophobic silver nanowires were dissolved in ethanol and chloroform, respectively. Different volumes of nanowire solutions (0.2 mg/mL) were sprayed onto PC substrates using a commercial airbrush fed with nitrogen. Substrates were placed on a hot plate at 120 °C for instant removal of solvents. In this deposition technique, the gas pressure and the distance between the airbrush and the substrate are crucial parameters to obtain reproducible and uniform films. The most homogeneous and reproducible films were obtained by keeping the nitrogen pressure constant at 29.7 psi and the nozzle-PC distance at 10 cm. This result is in agreement with the recent work published by Scardaci *et al*. [25].

Pressure-area isotherms of silver nanowires were recorded on the Langmuir Mini-trough (KSV, Espoo, Finland) placed on an anti-vibration table. Surface pressure was measured with a Pt-Wilhelmy plate connected to an electrobalance. The temperature at subphase, 20.0 ± 0.1 °C, was maintained by flowing thermostated water through jackets at the bottom of the trough. The temperature close to the surface was measured with a calibrated sensor from KSV, while the water temperature was controlled by means of a thermostat/cryostat Lauda Ecoline RE-106. The spreading solutions (0.75 mg/mL) were prepared using chloroform as solvent and deposited onto the water subphase with a Hamilton microsyringe. The syringe precision was 5 µL. Hydrophobic silver nanowires were transferred from the air-water interface onto PC by symmetric barrier compression (10 mm/min) with the substrate into the trough by horizontal dipping at 2 mm/min using a KSV2000 System 2 and the Holder model KN 0006 from KSV. To build nanowire networks with ordered arrangements, the LS films consist of two layers that are deposited in perpendicular orientation.

To improve the resistance of films by fusing nanowires [13], all electrodes were kept in an oven for three hours. Intensity-Voltage curves were measured using two tips over the electrodes with the Keysight B1500 Semiconductor Device, finding an Ohmic behavior in all electrodes. Voltages in a range from 50 mV to 1 V were applied and *R*_s_ values were obtained from the slope of the *I*-*V* curve. Transmittance and reflectance were also measured with a commercial Integrating Sphere equipped with Hg, Xe and Hg(Xe) DC arc lamps and a MS 257 spectrograph from Newport Corp.

The morphology of nanowires and electrodes were characterized using a Sigma HD Zeiss Field Emission Scanning Electron Microscopy (FE-SEM). The FE-SEM images were recorded by using accelerating voltages in the range from 1 kV to 10 kV.

## 4. Conclusions

We can modulate the sheet resistance and transmittance of AgNW films by modifying the capping agent and the deposition methodology. Our results proved that the replacement of the polymer PVP by alkyl thiol molecules does not modify the nanowire length and diameter; however, it does affect the lateral interactions between nanowires. Thus, when the hydrocarbon chain of alkyl thiol increases, the attractive interactions between them increase, promoting lateral aggregation. We proved that lateral aggregation decreases the electric resistance of nanowires and, consequently, it should be avoided. We achieved the best optoelectronic properties using PVP and C8-thiol as capping agents, which yield the highest percentages of non-aggregated nanowires. We also demonstrated that the spray-coating methodology is a good technique to obtain good quality AgNW films, while the Langmuir-Schaefer methodology allows for systematic modification of film transmittance, keeping resistance constant, or the sheet resistance, keeping film transparency constant. Finally, we proved that nanowires stabilized with PVP or octyl thiol can be employed as substitutes of ITO as a component of devices such as touch screens, electromagnetic shielding, and defrosted windows.

## Supplementary Materials

Supplementary materials can be accessed at: www.mdpi.com/1996-1944/8/11/5405/s1.
