# Supplementary material for: Modulating the Optoelectronic Properties of Silver Nanowires Films: Effect of Capping Agent and Deposition Technique
Source: Materials (Basel). 2015 Nov 11;8(11):7622–33. doi: 10.3390/ma8115405 (PMC5458922; doi:10.3390/ma8115405)
Supplement: Supplementary file 1 [file materials-08-05405-s001.pdf]

Supplementary Materials

1. Nanowire Characterization

1.1. UV-Vis Spectroscopy

UV-Vis spectra of AgNWs dissolved in ethanol (PVP) and chloroform (C8-C18) have been recorded using a Shimadzu UV-2401PC spectrophotometer and they are shown in Figure S1. As can be seen, all spectra present an intense band around 400 nm. This band is characteristic of transversal Surface Plasmon Resonance of AgNW and it is related with the diameter of AgNWs. Hence, the position of the maximum is almost independent on the nature of molecules used as capping agents; the diameter is not modified when the capped agent is exchanged.

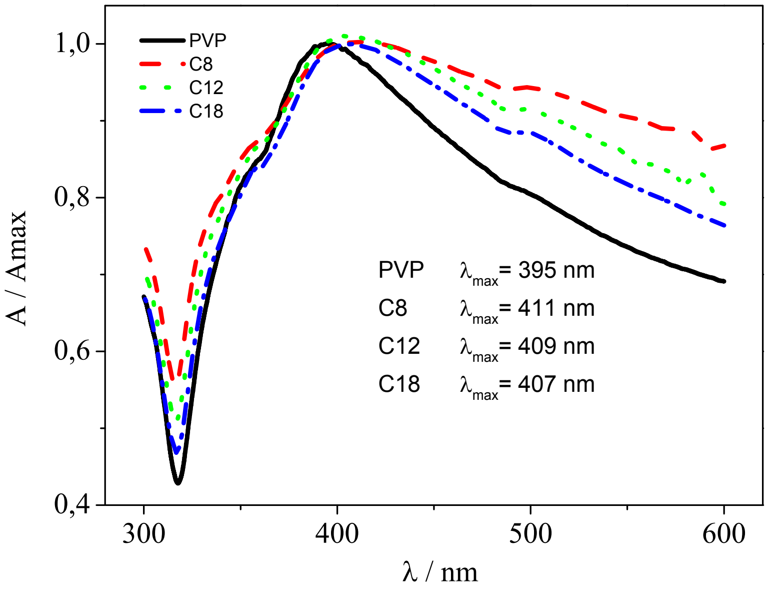

Figure S1. Normalized UV-Vis spectrum of AgNW solutions.

1.2. Length and Diameter Characterization. FE-SEM

To characterize the length and diameter, nanowires were spin coated onto Si/SiO_2 at 1000 rpm. The nanowire solutions were prepared on ethanol or chloroform depending on the nanowire nature. The solution concentration was kept constant at 0.2 mg/mL. FE-SEM images are collected in Figure S2.

FE-SEM images show the existence of single and laterally fused nanowires. The statistical analysis of FE-SEM images was carried out using the Smart SEM Zeiss Software. At least 50 nanowires have been measured to obtain representative statistical data. These results are shown in Figures S3 and S4. The length distribution was fitted to a Gaussian function in which the center represents the major contribution and the full width at half maximum, FWHM, was taken as an estimate of the polydispersity degree.

The statistical analysis of the diameter values revealed at least three different distributions; the center and the percentage of each one are represented in Figure S4. All results are summarized in Table S1.

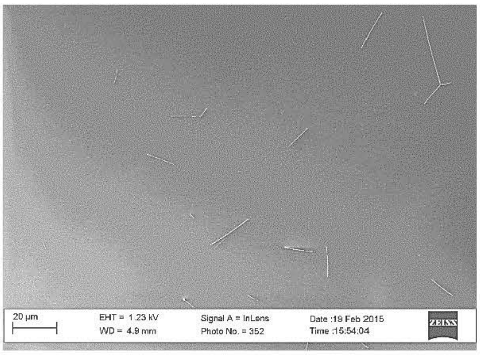

PVP-AgNW

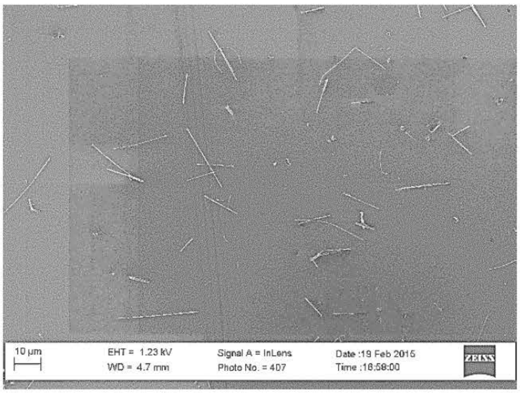

C12-AgNW

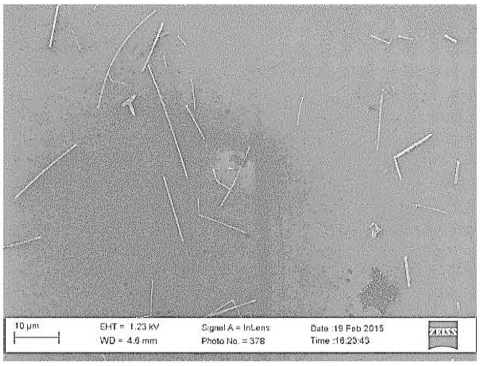

C8-AgNW

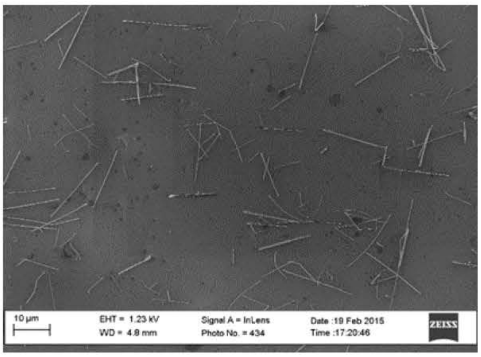

C18-AgNW

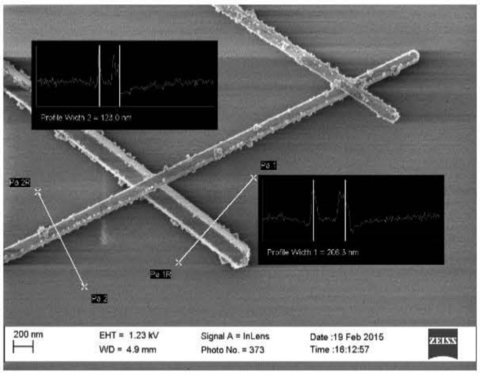

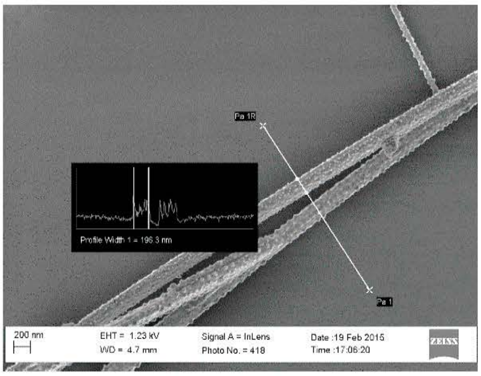

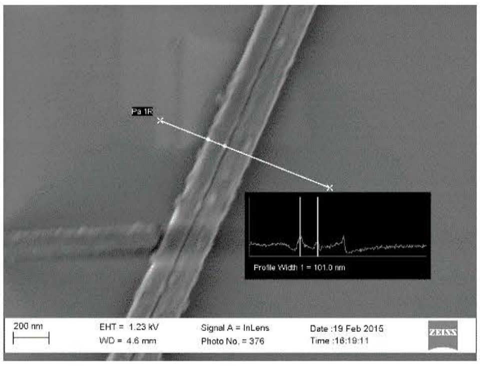

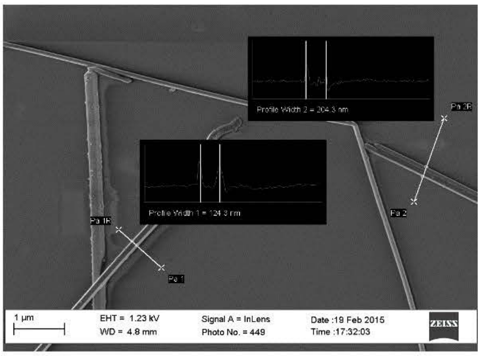

Figure S2. FE-SEM images of AgNW films at different magnifications.

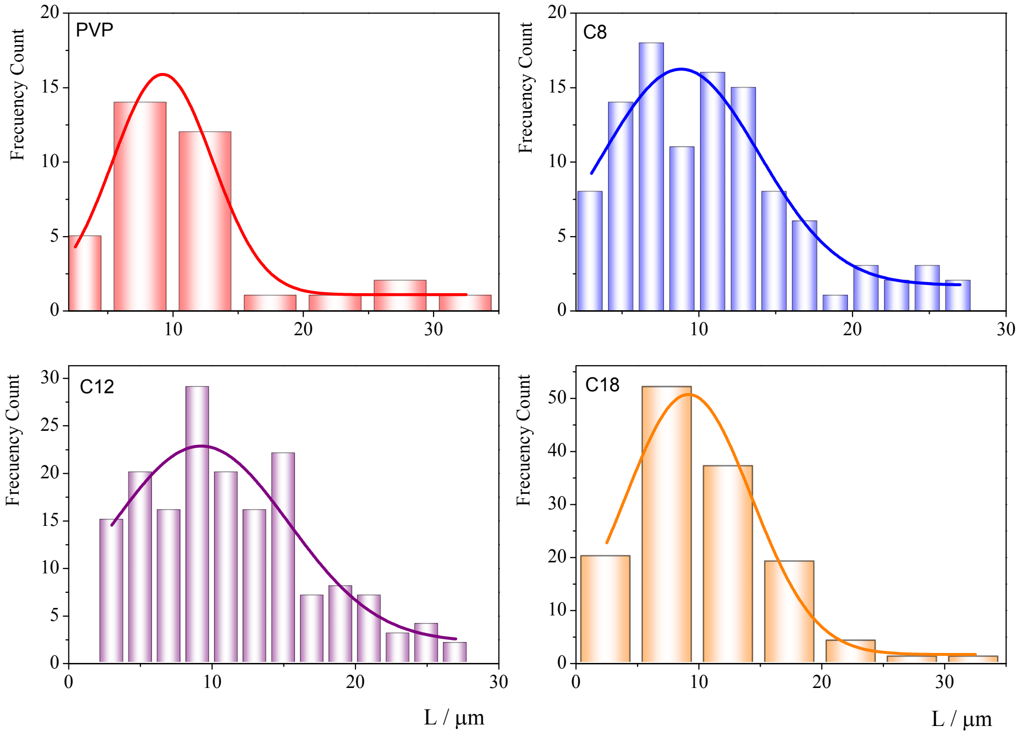

Figure S3. Length distribution of silver nanowires stabilized by PVP and alkyl thiol molecules (C8–C18).

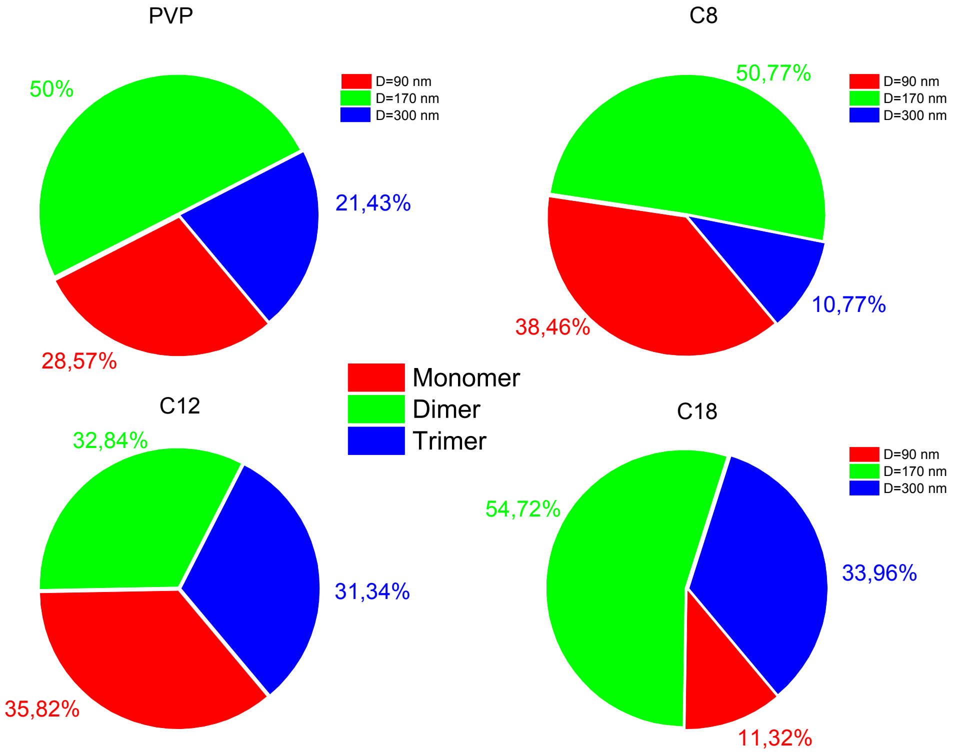

Figure S4. Nanowire diameter values and percentage of monomers, dimers and trimers for commercial AgNW (PVP) and modified with hydrophobic molecules (C_n-SH).

Table S1. Nanowire characterization obtained from the statistical analysis of FE-SEM images.

Capping Agent	*L*/µm	FWHM/µm	*D*/nm Single AgNW	Percentage of Single AgNW (%)	*D*/nm AgNW Aggregates	Percentage AgNW Aggregates (%)
PVP	9.2 ± 0.3	9 ± 1	83 ± 2	28.6	155 ± 16	50.0
					250 ± 5	21.4
C8	8.8 ± 0.7	12 ± 2	94 ± 6	38.4	160 ± 12	50.8
					254 ± 0	10.8
C12	9.2 ± 0.9	14 ± 4	89 ± 7	35.8	157 ± 5	32.8
					256 ± 12	31.4
C18	9.2 ± 0.5	12 ± 2	100 ± 0	11.3	160 ± 0	54.7
					327 ± 46	34.0

As can be seen from results in Table S1 the nanowire length, L, is almost independent on the capping agent being the averaged value of 9.1 ± 0.2 µm. The polydispersity degree is slightly higher for nanowires capped with alkyl thiol than for PVP-capped nanowires.

The diameter of single nanowires seems to be independent on the capping agent and the average value found was 92 ± 7 nm for monomers, 158 ± 3 for dimers and 270 ± 40. In addition, the percentage of single nanowires decreases when the number of C atoms of the alkyl thiol chain increases. This is an expected behavior if one considers that when the chain length of the capping agent increases, attractive interactions between capping agents also increase promoting lateral aggregation.

2. Nanowire Films

2.1. Experimental Conditions for Spray Deposition Of Nanowires

Different volumes of nanowire solution were deposited onto PC substrates positioned onto a hot plate at 120 °C by using commercial airbrush fed with nitrogen keeping the pressure constant at 29.7 psi and the nozzle-PC distance at 10 cm.

Results of Transmittance (*T*) and Sheet Resistance (R_s) for different films are collected in Figure S5 and Table S2.

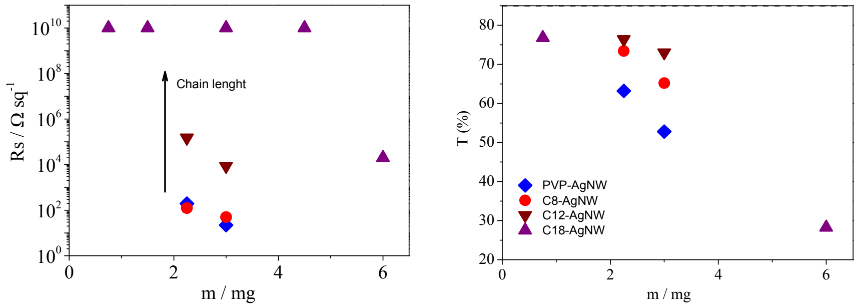

(**a**) (**b**)

Figure S5. Sheet resistance (**a**) and Transmittance (**b**) as a function of the sprayed mass in electrodes manufactured with different kind of AgNWs.

Table S2. Resistance and transmittance values of electrodes built with AgNWs, by spray-coating methodology.

Capping Agent	m/mg	*R*/Ω sq^{-1}	*T* (%)
PVP	2.25	192	63.2
	3.00	22	52.8
C8	2.25	19	73.4
	3.00	22	65.2
C12	2.25	1.5 10^5	76.4
	3.00	8580	73.0
C18	3.00	Insulator	56.0
	4.50	Insulator	40.3
	6.00	2.0 10^4	28.3

The sheet resistance corresponding to films built with C12-AgNW and C18-AgNW nanowires is too high and consequently, they do not seem a good choice for good transparent conductors. Therefore, we will have focused in AgNWs stabilized with PVP (PVP-AgNW) and octyl thiol (C8-AgNW).

2.2. Characterization of Thin Films Built by Spray-Coating Methodology

Figure S6 shows FE-SEM images of electrodes manufacturer with PVP-AgNW and C8-AgNW. The images correspond to films of different mass. Figure S7 shows pictures taken of these electrodes.

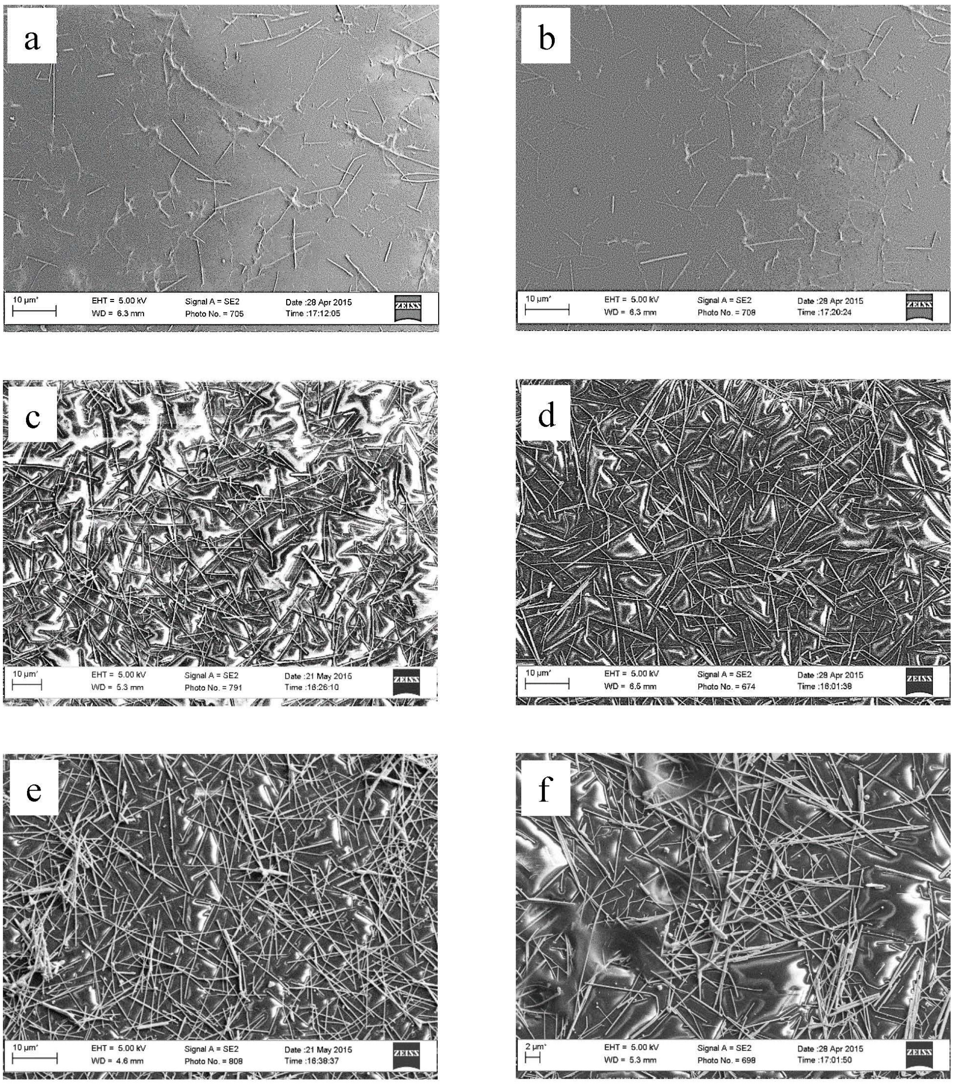

PVP-AgNW C8-AgNW

Figure S6. FE-SEM images of PVP-AgNWs (**left**) and C8-AgNWs (**left**) films prepared with different sprayed mass: 0.2 mg (**a**,**b**) 1 mg (**c**,**d**) and 2 mg (**e**,**f**).

From up to down in Figure S7, the nanowire density increases decreasing the transparency.

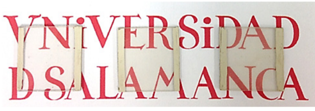

Figure S7. Pictures taken to C8-AgNW electrodes with different nanowire mass, from left to right: 0.2 mg, 1 mg and 2 mg.

2.3. Characterization of Thin Films Prepared by the Langmuir Schaefer Methodology

Figure S8 shows the FE-SEM image of a C8-AgNW film obtained by transferring the C8-AgNW monolayer at the surface pressure of 55 mN·m^{-1} from the air-water interface to the substrate. The film consists of two layers deposited in perpendicular orientation.

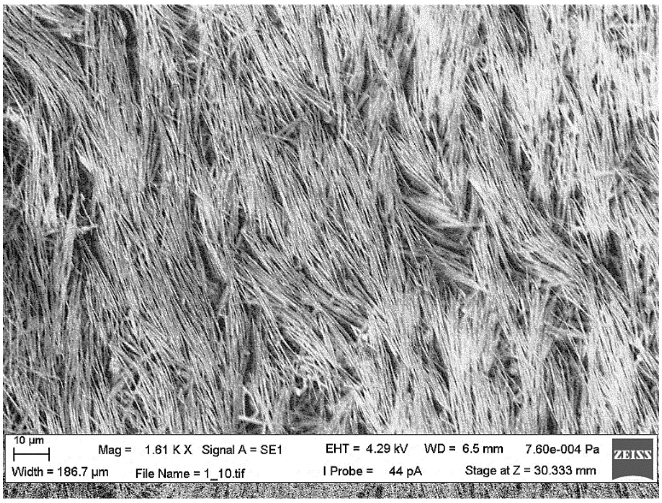

Figure S8. FE-SEM-image of the thin film built with two layers of C8-AgNW, see text.

Figure S9 shows the FE-SEM images of bilayers corresponding to 2D-Gas state (a, b) and to Liquid Expanded State (c, d).

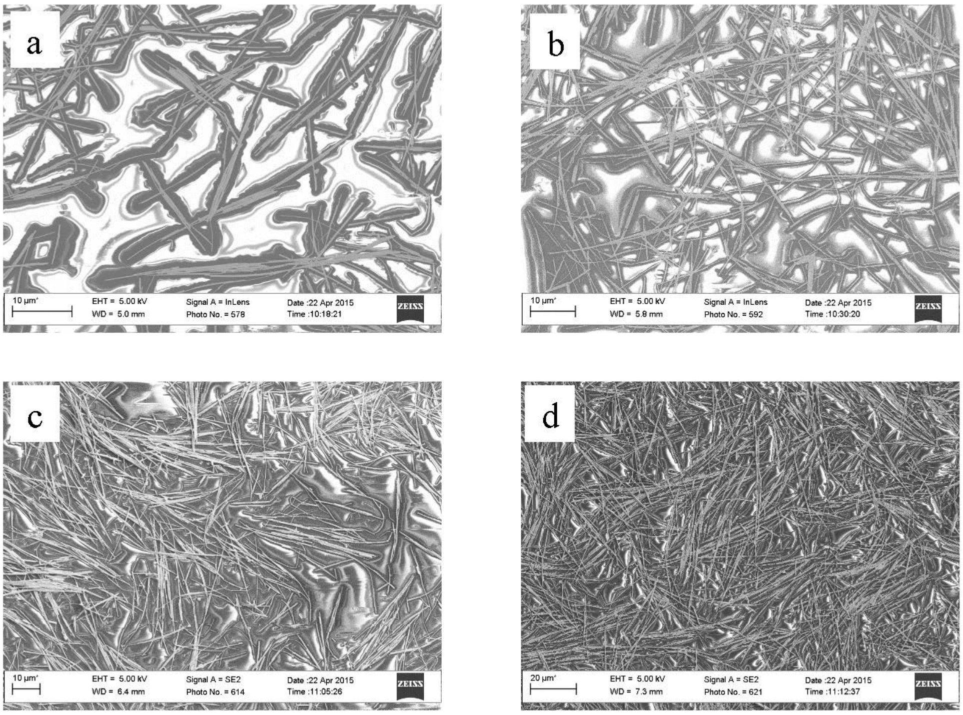

Figure S9. FE-SEM images of C8-AgNW bilayers deposited by the LS methodology at different surface pressures: (**a**) π = 0.3 mN·m^{-1} (Gas-state); (**b**) π = 2.5 mN·m^{-1} (Gas-state); (**c**) π = 5.0 mN·m^{-1} (LE-state) and (**d**) π = 20 mN·m^{-1} (LE-state).
